# Brief Motivational Interviewing in Dental Practice

**DOI:** 10.3390/dj7020051

**Published:** 2019-05-01

**Authors:** David G. Gillam, Huda Yusuf

**Affiliations:** 1 Oral Bioengineering, Institute of Dentistry, Barts and the London School of Medicine and Dentistry, Queen Mary University, London E1 2AD, UK; 2 Dental Public Health and Primary Care, Institute of Dentistry, Barts and the London School of Medicine and Dentistry, Queen Mary University, London E1 2AD, UK; h.yusuf@qmul.ac.uk

**Keywords:** motivational interviewing, dental practice, patient-centered, patient motivation, brief behavior change intervention

## Abstract

Motivational Interviewing has been demonstrated to be effective for a wide range of health behaviors. It is an effective behavior change method, which can be utilized in the dental practice setting. It can be used as a brief intervention to increase motivation to improve patients’ oral hygiene behaviors as well as providing a framework for delivering diet, smoking cessation, oral health changes, and alcohol advice. It involves four processes: engaging, focusing, evoking, and planning, guiding, which supports the patient towards a positive behavior change. Motivational Interviewing is a collaborative, patient-centered approach evoking the patient’s own motivation to change, thereby enhancing the relationship between the clinician and patient and improving patient outcomes. This review will provide an overview on the topic for dental professionals as well as helpful suggestions for supporting a positive behavior change in their dental practices.

## 1. Introduction

Dental teams are ideally positioned to provide both preventive advice and brief interventions to benefit patients, beyond the remit of solely improving their oral health, but also tackling chronic disease conditions including obesity, Type II diabetes, cardiovascular diseases, and cancers. It is important, however, to acknowledge that there are several clinician patient-related and environmental barriers to providing health-promoting advice in a dental setting. Although dentists acknowledge that there are opportunities for both prevention and reducing health risk behaviors associated with chronic diseases [1], there are also barriers to adopting a preventive approach such as a lack of financial incentives and a lack of time and training, in addition to concerns about upsetting the dentist–patient relationship [2,3]. It is well recognized that there is variation in the provision of clinical care and the implementation of evidence-based guidelines in the dental practice setting by health professionals [4]. This is because the behavior of healthcare professionals is determined by a series of individual factors such as: individual knowledge, skills, attitudes, motivation, cognitions and confidence. Another important factor is the organization of the dental practice (resources, personnel, skill-mix), which is in turn influenced by wider factors such as economic, political, and healthcare environments [4,5]. For example, guidance from the National Institute of Health and Care Excellence in the UK entitled “Oral Health: Approaches for general practice teams on promoting oral health (2017)” includes ample evidence on the psychological behavior change models which positively improve patient knowledge and influence oral hygiene behaviors and gingival health [6]. Furthermore, there is moderate evidence that patient motivation is partially dependent on the dental team’s communication skills and ability to build rapport with their patients [6], although they may not always adhere to the health advice provided by healthcare professionals [7]. Behavior(s) of patients is determined by a number of factors including attitudes, beliefs, social norms, and perceived control [7]. Nevertheless, behavior change is complex and there is limited understanding of how these elements interact to achieve the optimal behavior change between health professionals and their patients.

Traditionally, behavior change interventions have focused on clinicians exerting their influence on patients to change their behaviors without considering factors such as patient motivation or ambivalence. On a micro-level, behavior change in a clinical setting is influenced by the doctor–patient interaction, and the patient attempting to exert their autonomy by resisting change in their behaviors [8]. Hence, evidence has emerged that the more patient-centered approaches produce better outcomes [9]. Several counselling methods have been based on different psychological models and have been applied in a range of health care settings including cognitive therapy, behavioral therapy, cognitive behavioral therapy (CBT), advice, and feedback [10,11,12]. The choice of behavior method is, however, reliant on several factors such as the context (primary or secondary/hospital care), the target group (patient) and the outcome (improvements in oral hygiene, reduction in sugars, smoking cessation, and alcohol reduction) all of which have an important bearing on the choice of the health behavior change method *per se*. Motivational Interviewing (MI) has a number of characteristics that correspond to these requirements such as: (1) MI can be utilized as a brief intervention delivered in a resource- and time-constrained environment such as in general dental practice; (2) MI is client-centered, respecting client autonomy [8]; and (3) the evidence from systematic reviews has demonstrated that MI is effective in promoting a number of behaviors relevant to dental practice [13].

## 2. Why Use Motivational Interviewing (MI) in Clinical Practice?

MI offers a therapeutic approach for helping individuals increase their motivation or ‘readiness’ to change [14]. A patient-centered approach is more likely to result in a patient taking positive actions towards behavior change, which would also improve patient satisfaction with the dental consultation. From a communication perspective, the dentist or allied health professional can work collaboratively towards positive behavior change. This may mean that the clinician rather than telling the patient what to do, simply guides them in their decision and supports them towards an achievable goal. The clinician’s role is therefore collaborative in approach, evoking and eliciting from the patient motivation to change while having respect for patient autonomy.

### 2.1. Is MI Effective?

The main success of MI has been demonstrated in the addiction field, particularly in alcoholism and smoking cessation [15,16]. However, MI has been used in over 200 randomized clinical trials [RCTs] since the 1990s for a range of health behaviors including diabetes, pain management, eating disorders, dietary change, and oral health [13]. Numerous systematic reviews have also been conducted on the efficacy and effectiveness of MI across a range of behaviors. One of the first meta-analyses assessed the efficacy of MI and included 30 controlled trials including drug use, alcohol, HIV risk, diet, and exercise [17]. The analysis showed that adaptations to MI (AMIs) had moderate effects (0.25–0.57) compared with the control group in the areas of diet and exercise and these effects appeared to be sustained at follow-up up to 4 years after the treatment. Subsequently, a second meta-analysis which included 72 randomized controlled trials using MI for substance abuse, diet and exercise was shown to be effective [18]. A review of four meta-analyses also demonstrated that MI was significantly (10%–20%) more effective at reducing risky health behaviors than ‘no treatment’ controls across multiple behaviors including substance use of alcohol, marijuana, tobacco, and other drugs [19].

MI has also been adapted to provide a brief intervention in various healthcare settings ranging from 5 to 20 min. For example, Senft et al. [20] developed a brief 10-min intervention among heavy drinkers, whereas other researchers targeted smoking cessation [20,21]. Butler et al. developed a 5- to 10-min smoking intervention, which focused on quitting and the confidence to succeed [21]. The use of a brief version of MI may, however, be more feasible in dental practices where time constraints are inherent to daily practice. A systematic review assessed the effectiveness of MI on improving a range of oral health behaviors and clinical outcomes such as dental caries and periodontal outcomes. The authors identified 10 intervention studies, four of which were conducted in specific immigrant and low-income populations aimed at parents of younger children [22]. In the selected studies, the number of sessions ranged between 1 to 7 sessions with a duration of 15 to 45 min. Heterogeneity in the study design did not, however, allow for a quantitative assessment of the impact of MI on oral health outcomes. Although MI had no effect on changing behaviors in relation to oral hygiene in child populations, it did have a positive effect on parents who were advised to take their children to the dentist for fluoride varnish applications. However, it was noted that better quality studies are required, including the assessment of the optimal dosage of MI to demonstrate impacts on oral health.

The effectiveness of MI in changing oral health behaviors and oral health was compared with conventional health education approaches in a systematic review, which included 16 studies [23]. Among seven studies with the aim of improving periodontal health, MI outperformed the traditional methods of health education in five out of the seven studies. There were no significant differences in plaque reduction between those in the MI group vs. the health education group. However, the decrease in gingivitis was sustained for a longer period in the MI group (6 months) vs. the conventional group (1 month). A systematic review, which assessed the effects of MI as an adjunct to periodontal therapy also, showed some promising results in relation to the clinical periodontal parameters (plaque values, gingival, and periodontal inflammation) in two out of the four studies [24].

### 2.2. What Is MI?

MI was originally defined as a client-centered counselling approach for eliciting behavior change by helping clients to explore and resolve ambivalence [14]. The definition of MI was further revised in 2009 as “a collaborative person-centered form of guiding to elicit and strengthen motivation for change”.

Motivation has been defined as the energy and persistence that directs behaviors [25]. Miller conceptualizes motivation as a state of readiness to change, rather than a personality trait [26]. As a state, motivation may fluctuate over time or from one situation to another. Thus, a lack of motivation is not seen as inherent, but rather as something that is amenable to change. The primary focus of MI is therefore to facilitate behavior change by exploring and resolving ambivalence about behavior change.

A patient-centered approach is more likely to result in a patient taking positive actions towards behavior change, which would also improve patient satisfaction with the dental consultation. From a communication perspective, the dentist or allied health professional can work collaboratively towards positive behavior change. 

## 3. MI in Practice

The spirit of MI as a concept is a guiding clinical style that allows patients to reflect on their own motivation to change [26]. This goes beyond how we communicate with patients verbally, but also in terms of our attitudes, body language and facial expressions.

The Spirit of MI includes four aspects [26]:

**Partnership**: working together with equal input from patient and clinician, respecting patient autonomy.

**Acceptance**: understanding the patient’s perspective and not being judgmental; ensuring that we focus on their positive health behaviors and support them with their self-efficacy to change unhealthy behaviors.

**Compassion**: supporting patients who may be struggling with behavior change, showing real compassion and empathy towards them.

**Evocation**: is an attempt to explore what is important to the patient and how they would like to make a change.

There are several skills and methods used to communicate effectively with patients, which are included in the Table 1 below [27]:

It is important to exchange information in a collaborative rather than in an imposing manner [28]. As clinicians, we can feel compelled to offer advice in the first instance because of our duty of care to the patient. Instead, the clinician can offer information with permission from the patient, thereby respecting patient autonomy. Information should be offered in the spirit of trying to support patients in making informed decisions about their health.

**Figure 1:**
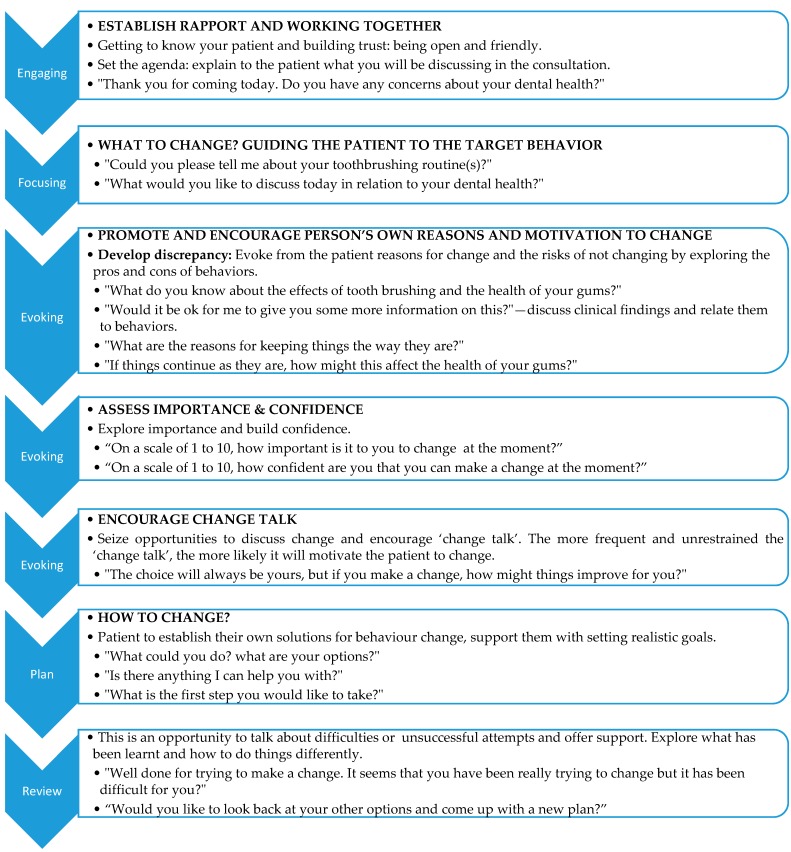
Steps in Motivational Interviewing—going back and forth in steps according to individual patients’ needs.

Tackling each behavior at a time in relation to oral hygiene as an example may be helpful in setting the agenda. According to the philosophy of motivational interviewing, it would be inappropriate to provide a prescriptive program of sessions for the clinician to adhere to. A flexible approach must be utilized. This is partly due to different patients being at different levels of readiness. There are several steps, which need to be considered: engaging, focusing, evoking, planning, and review (Figure 1), which has been adapted from Miller and Rollnick [28].

Recognizing that patients may have different levels of motivation to change, various strategies need to be employed by tailoring the intervention according to patient’s readiness to change. Patients can be generally categorized into (1) those not ready for change, (2) those who may be ambivalent or unsure about change, and (3) those who are ready to change [8,29]. For example, if the patient is not ready to change, there is little merit in trying to persuade the patient to change. The clinician’s role is to respect the patient’s autonomy and acknowledge that they are not ready to change. The clinician can also raise awareness about current behaviors and offer an exchange of information with the patient’s permission. It is important to ask the patient whether it is acceptable to bring up the subject of behavior change at their next appointment. This is because motivation to change is not static and may fluctuate with time. If the patient is unsure, the clinician’s role is to explore ambivalence further and build on the motivation and confidence to change [8]. This means exploring the pros and cons of current behaviors, engaging in reflective listening, and summarizing the argument for and against behavior change. If the patient is ready to change, the clinician’s role is to build on the commitment and motivation to change and ask the patient to come up with feasible steps/goals to make change.

There are four processes involved in MI: engaging, focusing, evoking, and planning and as such are intended to be used as a guide during the consultation (Figure 1). In other words, it is not necessary to include all four processes in the consultation with patients and this does not occur in a linear order [28]. The processes are therefore fluid and are not meant to be formulaic in nature.

### 3.1. Engaging

The first step is to engage with the patient and build a rapport establishing an open and trusting relationship between the clinician and patient. It is important to show empathy from the outset to support a collaborative approach to behavior change. This includes reflective listening and understanding of what is going on for the patient and understanding their perspective. This is followed by agenda setting and explaining to the patient what will be discussed in the consultation as part of planning for the session.

Establishing Rapport: “a helpful connection, a collaborative working relationship”.

### 3.2. Focusing

This phase is important in identifying the target behavior ‘what to change’. Focusing is the process by which a clinician in collaboration with the patient, develops and maintains a specific direction in the conversation about change. For example, asking the patient whether they would be happy to discuss smoking cessation or bleeding gums in relation to their periodontal health. This is not static and the focus or re-focus can change over time.

Examples can include:

“It seems you are concerned about your gums bleeding today. Would you be happy to discuss this now?”

“Should we try to find out what is the cause of your gums bleeding?”

### 3.3. Evoking

This is about getting the patient to identify their intrinsic motivation to change and coming up with their own ideas and plans towards behavior change.

A first step may be to gauge the patients’ knowledge and understanding on maintaining healthy gums. This helps the clinician to identify any gaps in their knowledge and understand their skills, which may affect the patient’s motivation to change. The role of the clinician is therefore to fill in the gaps and provide the patient with the relevant information after seeking their permission. It is important to seek permission, so the patient does not perceive this as ‘telling them what to do’. The information should be given in the right circumstances especially if the patient asks for it.

Examples can include:

“You seem to have a good awareness of what may affect the health of your gums and teeth. Would it be ok for me to tell you more about this? There are a couple of comments I could make…”

During this step, both ambivalence and discord (defensiveness, arguing, ignoring) may occur. If a patient refuses to take up the offer of information exchange at the initial appointment because they may not be ready, you could ask the patient “is it ok for me to discuss this with you at our next appointment”. The ethos of MI is to ask permission before providing unsolicited information.

It is important to listen to the patient and look out for change talk. Change talk is defined as patients’ statements about their desire or reasons or need to change. This is pivotal, as evidence has shown that change talk is associated with enhanced motivation for change, which is in turn associated with increased likelihood of actual change [26]. The role of the clinician is to listen for and elicit change talk from the patient. Additionally, it is the clinician’s role to highlight the pros and cons of the patient’s oral hygiene behaviors and he/she should evoke from the patient the reasons for any proposed change and risks of not changing. It can also include assessing how important it is for the patient to make a change and how confident they feel in attempting to change a behavior.

Reflective listening is important at this stage to decrease the likelihood of misinterpreting what the patient is telling the clinician and clarifying the patient’s thinking. This can be accomplished through paraphrasing or repeating and reflecting the feelings and thoughts of the patient. Using summation and reflective listening can also help articulate any contradictions between the clinician and the patient.

The patient may also express their concerns and may start talking about behavior change. The clinician can take such opportunities to discuss change and encourage ‘change talk’. The more frequent and unrestrained the ‘change talk’, the more likely it will influence patient behaviors.

“It sounds like that you would like to stop smoking but you can’t at this stage…” Is this right?

(Clarify what they have said)

The next step is to explore and clarify their thoughts.

“It seems that you would like to stop smoking but you feel you are not ready at this stage”

“If things continue as they are, how might this affect the health of your gums?”

“On a scale of 1 to 10, how confident are you that you could make this change if you wanted to?”

### 3.4. Planning:

This step involves the patients finding their own solutions for behavior change. The clinician’s role is to help the patient develop realistic and achievable goals and strategies for how to achieve them. It is important that the patient commits to change rather than the clinician telling the patient how to change. The patient therefore has to believe in his or her own argument(s) for change, which in turn will increase the likelihood of behavior change.

- “Is there anything I can help you with?”
- “What is the first step you would like to take?”

### 3.5. Review

Reviewing the patient in subsequent visits allows the patient to provide any feedback to the clinician. At this point, both the clinician and patient can explore any challenges and facilitators to behavior change. The patient’s knowledge in attempting any behavior change should be acknowledged and the patient should be congratulated for attempting to change. This builds on both self-efficacy and confidence. Any failures should be revisited, exploring the reasons for failure to develop an alternative action plan.

## 4. Discussion

Traditionally, chairside patient education has focused on the clinician exerting their expertise on patients. In the case of patients failing to comply, clinicians feel frustrated and may lead to ‘victim blaming’ the patient. Patient-centered methods provide a collaborative approach to behavior change in a safe, non-judgmental and supportive environment. Patients can take control of their behaviors and are therefore more likely to succeed in any subsequent behavior change. Motivational Interviewing is a useful method to promote behavior change as it can be used in dental practice settings. It offers an evidence-based approach to behavior change, which potentially improves patient–clinician interactions [30]. Unlike other behavior change methods such as CBT, which may require expertise, MI principles can be used to deliver brief interventions in dental practice settings. Motivational Interviewing allows the clinician to adapt their intervention according to the stage of readiness of the patient. It is especially appropriate in primary care settings as it can be as brief as traditional methods of health advice [18,30]. This is particularly relevant to dental practice as patients are usually seen regularly to maintain their oral health or may be receiving dental treatment over repeated visits. In studies where MI sessions lasted less than 20 min, 64% (7 out of 11 studies) showed a significant effect on behavior change. The likelihood of an effect increased with the number of encounters and a prolonged follow-up [18]. Overall, MI outperformed traditional advice in 75% of the studies and its effectiveness was not related to the counsellor’s educational background. The promising effectiveness of MI to promote behavior change can also impact on dental health education in dental schools. It is recognized that the dental curricula are limited in their teaching of behavior change methods. Implementing MI in the curriculum would enable students to benefit from learning new skills to support their patients to positive behavior change [31,32].

There are challenges in learning MI. Although in principle it may appear to be easy to learn, it does require a level of competence and time to transmit the training into practice [33]. It requires training and on-going support and supervision in order to master the method. Furthermore, there may be a potential conflict between the practice of MI and clinicians reverting to their traditional methods of health education [34].

## 5. Conclusions

Motivational Interviewing is an effective behavior change method, which can be utilized in the dental practice setting. It can be used as a brief intervention to motivate patients to improve their oral hygiene behaviors as well as providing a framework for delivering diet, smoking cessation, and alcohol advice. Motivational Interviewing can provide a collaborative approach to behavior change in a safe, non-judgmental and supportive environment to enable patients to take control of their behaviors. Although MI can be challenging to both the clinician and patient, it is a versatile method, which can be successfully utilized in the general dental practice setting.

Motivational Interviewing contributes to both shared decision-making and patient-centered care. MI is versatile and can be integrated into primary care, promoting healthy life styles as a brief intervention or it can be extended to offer a more comprehensive intervention for behavior change. It can also be used for a range of behaviors relevant to both oral and general health, thereby improving patient outcomes.

## 6. For Training on MI

There is an international organization known as Motivational Interviewing Network of Trainers, which provides a list of trainers and events in supporting the use of MI in clinical practice and research [35].

## Figures and Tables

**Table 1:** Skills and methods used to communicate effectively with patients (OARS).

OARS
Open-ended questions: it is important to use open-ended questions which facilitate a dialogue between the clinician and patient so that the patient is encouraged to speak and the clinician listens.
Affirmations: acknowledge that behavior change is difficult and promote patient self-efficacy by affirming their strengths.
Reflective listening: this is about listening and understanding what the patient has expressed. This can enhance the relationship between the clinician and the patient, encouraging them to continue exploring change in behaviors and supporting motivation.
Summarizing: this demonstrates that the clinician has listened to the patient and provides a concise overview of what has been discussed. This may also support dealing with ambivalence.
